# A comparative study of electrochemical oxidation of methidation organophosphorous pesticide on SnO_2_ and boron-doped diamond anodes

**DOI:** 10.1186/s13065-015-0136-x

**Published:** 2015-10-16

**Authors:** Fatima Hachami, Mohamed Errami, Lahcen Bazzi, Mustapha Hilali, Rachid Salghi, Shehdeh Jodeh, Belkheir Hammouti, Othman A. Hamed

**Affiliations:** Faculté des Sciences d’Agadir, Laboratoire Matériaux & Environnement, Equipe de Chimie Physique Appliquées, BP 8106, 80000 Agadir, Morocco; Ecole National des Sciences Appliquées d’Agadir, Laboratoire d’Ingénierie des Procédés de l’Energie & de l’Environnement, BP 1136, 80000 Agadir, Morocco; Laboratoire d’Innovation et Recherche Appliquée (LIRA), Ecole Polytechnique Université Internationale d’Agadir, 80000 Agadir, Morocco; Department of Chemistry, An-Najah National University, P. O. Box 7, Nablus, State of Palestine; LCAE-URAC18, Faculty of Sciences, Mohamed 1st University, 60000 Oujda, Morocco

**Keywords:** Electrooxidation, Energy consumption, Methidathion, BDD anode, SnO_2_ anode

## Abstract

**Background:**

Electrochemical oxidation considered to be among the best methods in waste water desalination and removing toxic metals and organic pesticides from wastewater like Methidathion. The objective of this work is to study the electrochemical oxidation of aqueous wastes containing Methidathion using boron doped diamond thin-film electrodes and SnO_2_, and to determine the calculated partial charge and frontier electron density parameters.

**Results:**

Electrolysis parameters such as current density, temperature, supporting electrolyte (NaCl) have been optimized. The influences of the electrode materials on methidathion degradation show that BDD is the best electrode material to oxidize this pesticide organophosphorous. Energetic cost has been determinate for all experiments. The results provide that 2 % of NaCl, 60 mA cm^−2^ and 25 ºC like the optimized values to carry out the treatment. For BDD the achieved Chemical Oxidation Demand reduction was about 85 %, while for SnO_2_ it was about 73 %. The BDD anode appears to be the more promising one for the effective electrochemical treatment of methidathion. Finally the theoretical calculation was done by using the calculation program Gaussian 03W, they are a permit to identify the phenomena engaged near the electrode and to completely determine the structures of the products of electrochemical oxidation formed during the degradation and which they are not quantifiable in experiments because of their high reactivity.

**Conclusions:**

The comparison of the results relating to the two electrodes indicates that these materials have a power to reduce the quantity of the organic matter in the electrolyzed solution. But the speed of oxidation of these compounds is different according to the materials of the electrodes used.

**Graphical abstract:** Structural of methidathion [O,O-dimethyl-S-(5-methoxy-1,3,4-thiadiazolinyl-3-methyl) dithiophosphate] used for study the electrochemical oxidation

## Background

Electrochemical oxidation considered to be among the best methods in waste water desalination and removing toxic metals and organic pesticides from wastewater like Methidathion [1]. The electrochemical reactions are difficult and need a lot of explanation. Most of the products are depending on the products of oxidation and free radicals. The electrochemical oxidation in wastewater using both SnO_2_ and BDD (boron-doped diamond) as anode goes in two steps [2]. The first one is the anodic discharge of the water (Eq. 1), in which the hydroxyl group radical adsorbed on the electrode surface (M [ ]) as shown in Eq. 2.

1 $${\text{H}}_{ 2} {\text{O }} + {\text{ M }}\left[ \, \right] \, \to {\text{ M}}\left[ {{\text{OH}}^{ - } } \right] \, + {\text{ H}}^{ + } + {\text{ e}}^{ - }$$

in which the hydroxyl radical oxidized the organic matter in wastewater.

2 $${\text{R }} + {\text{ M }}\left[ {{\text{OH}}^{ - } } \right] \, \to {\text{ M}}\left[ \, \right] \, + {\text{ RO }} + {\text{ H}}^{ + } + {\text{ e}}^{ - }$$

where RO is the oxidized organic matter. The radicals, ${\text{OH}} \cdot$, ${\text{O}} \cdot$ and ${\text{ClO}} \cdot$ have a very short life-time due to their high oxidation potential. Effective pollutant degradation depends on the direct electrochemical process due to the secondary oxidants which cannot convert all organics to water and carbon dioxide [1].

This study concentrates on understanding the behavior of degradation and understanding using BDD in degradation of some pesticides like Methidathion.

Recently, Errami and co works [3–6] demonstrated that the pesticides difenoconazol, bupirimate can be electrochemically removed from aqueous solutions using BDD anodes. They found that current density influence is remarkably clear on the BDD electrodes.

We have chosen to study the Methidathion as cited above because the pesticides residues analyses from 83 samples pick up from 20 packinghouses in the area of Souss Valley, in the southern part of Morocco, revealed that the compounds frequently found are Methidathion, Chloropyriphos ethyl, Malathion, Dimethoate and Parathion-methyl at a rate of 43, 33, 11, 7 and 4 % respectively of the number of samples [7, 8].

Methidathion [O,O-dimethyl-S-(5-methoxy-1,3,4-thiadiazolinyl-3-methyl) dithiophosphate] is a widely used organophosphorous insecticide, it was chosen as the target molecule for the present study by its biotoxicity (The acute oral LD50, for rats is approximately 54 mg/kg [9].

The experimental results have indicated that the efficiency of electrochemical oxidation of BDD is higher than that of SnO_2_ for the degradation of obsolete methidation organophosphorous pesticide stock. The electrochemical degradation mechanism of Methidathion was also discussed.

This paper reports the degradation of Methidathion solutions by electrochemical method such as anodic oxidation, with a SnO_2_ and boron-doped diamond (BDD) anode. Several techniques were proposed for the pesticides treatment. However the electrochemical oxidation is one of the best means in this field.

The objective of this work is to study the electrochemical oxidation of aqueous wastes containing Methidathion using boron doped diamond thin-film electrodes and SnO_2_, and to determine the calculated partial charge and frontier electron density parameters.

## Methods

### Chemicals

To understand the toxicity removal, several measurements of chemical oxygen demand (COD) has been done in triplicate and the three results where almost the same with 5 % differences.

The commercial formulation Methidaxide (40 % Methidation) was purchased from Bayer. Sodium chloride with high purity was purchased from Aldrich (Germany).

### Electrolytic system

The electrode BDD was synthesised using hot filimant chemical vapor deposition on conducting p-Si substrate (0.1 Ωcm, Siltronix).The filimant temperature was about 2500 °C while the substrate kept at 830 °C. The reactive gas used was methane in an excess of dihydrogene (1 % CH_4_ in H_2_). The doping gas was trimethylboron with a concentration of 3 ppm. The gas mixture was supplied to the reaction chamber, providing a 0.24 µm h^−1^ growth rate for the diamond layer. The diamond films were about 1 µm thick. This HF CVD process produces columnar, randomly textured, polycrystalline films.

SnO_2_ electrode is a commercial grid of surface equal to 1 cm^2^ (ECS International).

All electrochemical measurements (Cyclic voltammetry and galvanostatic electrolysis) were performed with a Potentiostat/Galvanostat PGP 201 associated to “Volta-Master1” software. A conventional 100 cm^3^ thermoregulated three electrodes glass cell was used (Tacussel Standard CEC/TH). Saturated calomel electrode (SCE) and platinum electrode are respectively, the reference and Auxiliary electrodes. The anode was a square plate of BDD electrode or SnO_2_ with effective surface area of 1 cm^2^.

Galvanostatic electrolysis experiments were carried out with a volume of 75 cm^3^ aqueous solution of Methidathion 1.4 mM during 120 min. The range of applied current density was 20–60 mA cm^−2^ and samples were taken, at predetermined intervals during the experiment, and submitted for analysis. All tests have been performed at different temperature in magnetically stirred and aerated solutions. In all cases sodium chloride was added to the electrolytic cell, at different concentrations.

### Analytical procedures

The Chemical Oxygen Demand (COD) values were determined by open reflux, a dichromate titration method. All chemicals used in the experiments were of analytical pure grade and used without further purification. All measurements were repeated in triplicate and all results were observed to be repeatable within a 5 % margin of experimental error. The UV–Vis spectra of Methidathion were recorded in 190–400 nm range using a UV–Vis spectrophotometer (UV-1700 Pharmaspec, Shimadzou) with a spectrometric quartz cell (1 cm path length). The method used for the extraction of methidathion was adapted from Charles and Raymond [10]. For each 5 mL of the sample, 100 mL of acetone was added and the mixture was stirred for 2 h. The extraction was carried out respectively with 100 and 50 mL of acetone. After filtration, the residues in acetone were partitioned with saturated aqueous NaCl (30 mL) and dichloromethane (70 mL) in a separating funnel. The dichloromethane fraction was collected and the separation process with (70 mL) dichloromethane were combined and dried over anhydrous sodium sulphate. The solvent was removed under reduced pressure at 40 °C and the residues were dissolved in an acetone-hexane (1:9) mixture (10 mL). Samples were analyzed by gas chromatography.

### Gas chromatography analysis

Analysis of the methidathion pesticide was carried out with a Hewlett–Packard 6890 gas chromatograph equipped with an NPD Detector, on-colum injection port, and HP-5 column (5 % diphenyl copolymer/95 % dimethylpolysiloxane) (25 m × 0.32 mm ID, 0.52 μm film thickness)and temperature programming from 80 to 160 °C at 25 °C/min. 220–240 °C at 10 °C/min, 80 °C (3.00 min), 160 °C (2.00 min), 220 °C (10.00 min), 240 °C (8.80 min); Injector temperature 73–250 °C (180 °C min). The temperature of the detector was 300 °C. Carrier gaz (helium) flow rate, 2.6 mL/min; makeup gaz (nitrogen) flow rate, 10 mL/min; Air 60 ml/min; H2 3 mL/min. The injection volume was 1 μL.

## Results and discussion

### Effect comparative study of electrochemical degradation efficiency on BDD and SnO_2_ electrodes

This paper presents a comparative study of the performances of two materials of electrodes, (BDD, SnO_2_) used in the same device under same conditions of electrolysis for the electrochemical oxidation of Methidathion. The electrodes of BDD and SnO_2_ were compared under same the operating conditions which had been fixed for the preceding experiments: the density of current imposed 60 mA/cm^2^, the temperature 25 °C, 2 % of NaCl and 1.4 mM of Methidathion.

### The Variation of the concentration

**Fig. 1:**
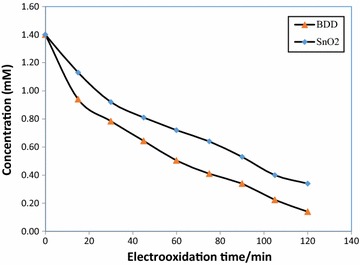
Electrolysis time dependence of methidation concentration for two anodes (BDD, SnO_2_). Methidation initial concentration = 1.4 mM, current density = 60 mA cm^−2^, electrolyte = 2 % NaCl)

The comparative study of electrochemical degradation of Methidathion was also performed on BDD and SnO_2_ electrodes. The concentration of Methidathion was measured using GC/NPD Detector; the variations of methidathion concentration with electrolysis time for the two anodes are shown in Fig. 1. However, the decrease trend was different on two electrodes. The changes in concentrations of the pesticide with the two electrodes, exhibit similar kinetic behavior. Indeed, during treatment, there is a decrease exponential and rapid concentration of pesticides to their virtual disappearance after 120 min by the electrode DDB by cons with SnO_2_ anode was a slowly decreasing the concentration of methidathion relative to that observed with the anode DDB. The concentration removal decrease from 90 % for BDD electrode to 72 % for SnO_2_ electrode the reaction rate is fast on the BDD anode, while the reaction rate is relatively slow on the SnO_2_ anode. These results show that the  % of abatement Methidathion found by GC is the same as analyzed by COD.

### The Variation of the COD and the abatement as a function of time

**Fig. 2:**
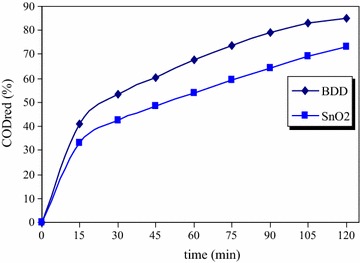
Rate of degradation of the Méthidathion in function to electrochemical time during treatments for electrode BDD and SnO_2_

The variation of the abatement in COD for electrochemical degradation of Methidathion is represented in Fig. 2. The electrolyses were realized in the optimal conditions for each electrode BDD and SnO_2_.

The variation of the abatement of COD as a function of time for the two electrodes BDD and SnO_2_ is represented in Fig. 2.

The result obtained to know the abatement in COD is more effective with BDD than with SnO_2_. The use of the BDD permits to attain the abatement in COD of 85 % whereas under the same conditions, SnO_2_ make it permit to attain 75 %. The efficiency of BDD is related to the capacity of produce hydroxyls radicals which are very powerful oxidants [11, 12].

**Fig. 3:**
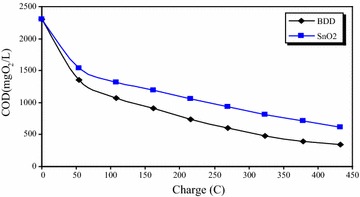
Evolution of the COD in function to the charge passed in the solution during the electrolyse

Figure 3 represents the variation of the COD as a function of the charge during the electrolysis of the solutions of Methidathion for the two anodic materials.

At the beginning of the electrolysis until a charge of 100 C, the oxidation of Methidathion is more rapid. After this charge, the curves of variation of the COD change slope, what indicates change of speed of production of the hydroxyls radicals.

**Fig. 4:**
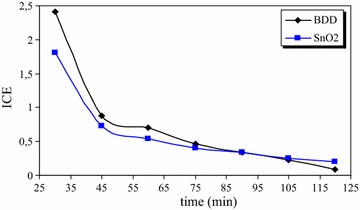
Variation of the instantaneous current efficiency during the electrolysis of a solution of Méthidathion 1.4 mM with the electrode of BDD and SnO_2_

The reaction of degradation of Methidathion is thus limited by the speed of the transfer of the charge. For a charge of 432 C the elimination of the COD for BDD and SnO_2_ respectively reached 345.6 mg/L and 612.7 mg/L. This indicates that the electrode of BDD is more effective than SnO_2_. These results are confirmed by instantaneous current efficiency represented in Fig. 4.

These curves representing ECI in function to time have permit to show that the electrode of DDB was more effective than the electrode of SnO_2_ with respect to electrochemical degradation of Méthidathion. The effectiveness of current decreases progressively with the time of electrolysis for the two anode materials, by gradual formation of products more difficult to oxidize [13, 14]. At the beginning of electrolysis, ECI >1, this can be interpreted by the chemical existence of phenomenon associated with the electrochemical reaction; this phenomenon has measurable effects only in the first moments of electrolysis [15].

### Energy consumption

There are two methods found in the literature to calculate the CE. The first method is the COD [11]. In this method the COD is measured at different time intervals. The Instantaneous current efficiency ICE. Is then calculated as:

$${\text{ICE}} = \frac{{\left( {\left( {\text{COD}} \right)_{t} - \left( {\text{COD}} \right)_{t + \Delta t} } \right)}}{8i\Delta t}{FV}$$

where (COD)_*t*_ and (COD)_*t*+1_ are the chemical oxygen demands (gO_2_ L^−1^) at times *t* and *t* +1 (s), respectively. I is the applied current (A), F the Faraday constant (96,487 Cmol^−1^) and V is the volume of the electrolyte (L).

This method could be misleading, since it measures the ICE with respect to the final product carbon dioxide.

**Fig. 5:**
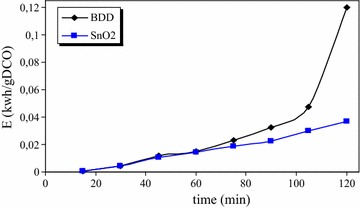
The variation of energy consumption with the electrodes BDD and SnO_2_ during 2 hours of treatment

From the energy point of view, the quantity of energy necessary during 2 h of electrolysis for two materials of anode is represented in Fig. 5. As can be seen from Fig. 5, the energy consumption at the beginning of electrolysis is approximation the same for the two electrodes. However, the abatement in COD is more significant for the electrode of BDD than SnO_2_. As well as the ECI at the first minutes of electrolysis, the diamond electrode for ECI was significant.

To destroy 73.4 % of the organic matter, the quantity of energy necessary is about 0.024 kWh/g COD for BDD pendant was 75 min, while with the SnO_2_ the necessary energy was about 0.037 kWh/g COD pendant and electrolysis the electrolysis time was about 2.0 h. The diamond electrode is thus more effective energetically than SnO_2_; this difference is related on the working time and to to the electrocatalytic activity.

The comparison of these materials of anode during electrolysis of Methidathion permit to conclude, not only that the electrode of BDD was more effective than the electrode of SnO_2_ opposite to the electrochemical degradation of Methidathion, but also it more effective energetically.

### The absorbance

**Fig. 6:**
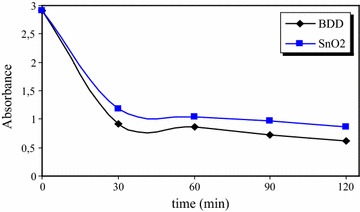
Evolution of the absorbance in function to time during the reaction of oxidation of Méthidathion for the electrodes BDD and SnO_2_

During the treatment of the solution of Methidation at a wavelength of 210 nm, the absorbance decrease in the course of the time of electrolysis for the two electrodes used BDD and SnO_2_; the results obtained are represented in Fig. 6. For each electrode the absorbance decreases quickly at the beginning of the electrolysis, this can be explained by the cleanliness of the surface of the electrode in the first minutes of treatment. Decrease in the rate of reduction with the time of electrolysis; can be explained by the adsorption of the organic Matter on the surface of the electrode what prevents the direct transfer of electrons between the studied molecule and the electrode.

The electrode BDD has an absorbance lower than that of the electrode of SnO_2_. Thus one can note that electrode BDD used under the conditions galvanostatic showed a great capacity to mineralize the organic compounds.

### Interpretation of the frontier electron density

Frontier electron densities and point charges were calculated using Gaussian 03 program. As summarized in Table 1, the results indicated that the most negative point charges were located on oxygen atoms O5, O4, O23 and O22 of −1.199251; −1.227905; −1.053448; −0.974324, respectively. Hence we could expect that the Méthidation could be adsorbed on the surface of the electrode maybe by oxygen port methyl at natural pH.

**Table 1:** Calculated partial charge and frontier electron density derived from RHF/6–31 + G (2d,2p) method

Atom	Partial charge	Frontier electron density	Atom	Partial charge	Frontier electron density
1P	2.346671	0.61767309	7N	−0.755026	0.05074912
2S	−0.764807	0.28262232	8N	−0.527338	0.00675071
3S	−0.519851	0.82272659	9C	1.139560	0.01041556
4O	−1.227905	0.15750226	0S	−0.182921	0.00631317
5O	−1.199251	0.11248018	1C	1.366795	0.02819456
6C	0.626992	0.00669293	2O	−0.974324	0.02423551
7C	0.571323	0.0022927	3O	−1.053448	0.00168228
14C	0.658733	0.95032112	4C	0.568562	2.5873E-05

**Fig. 7:**
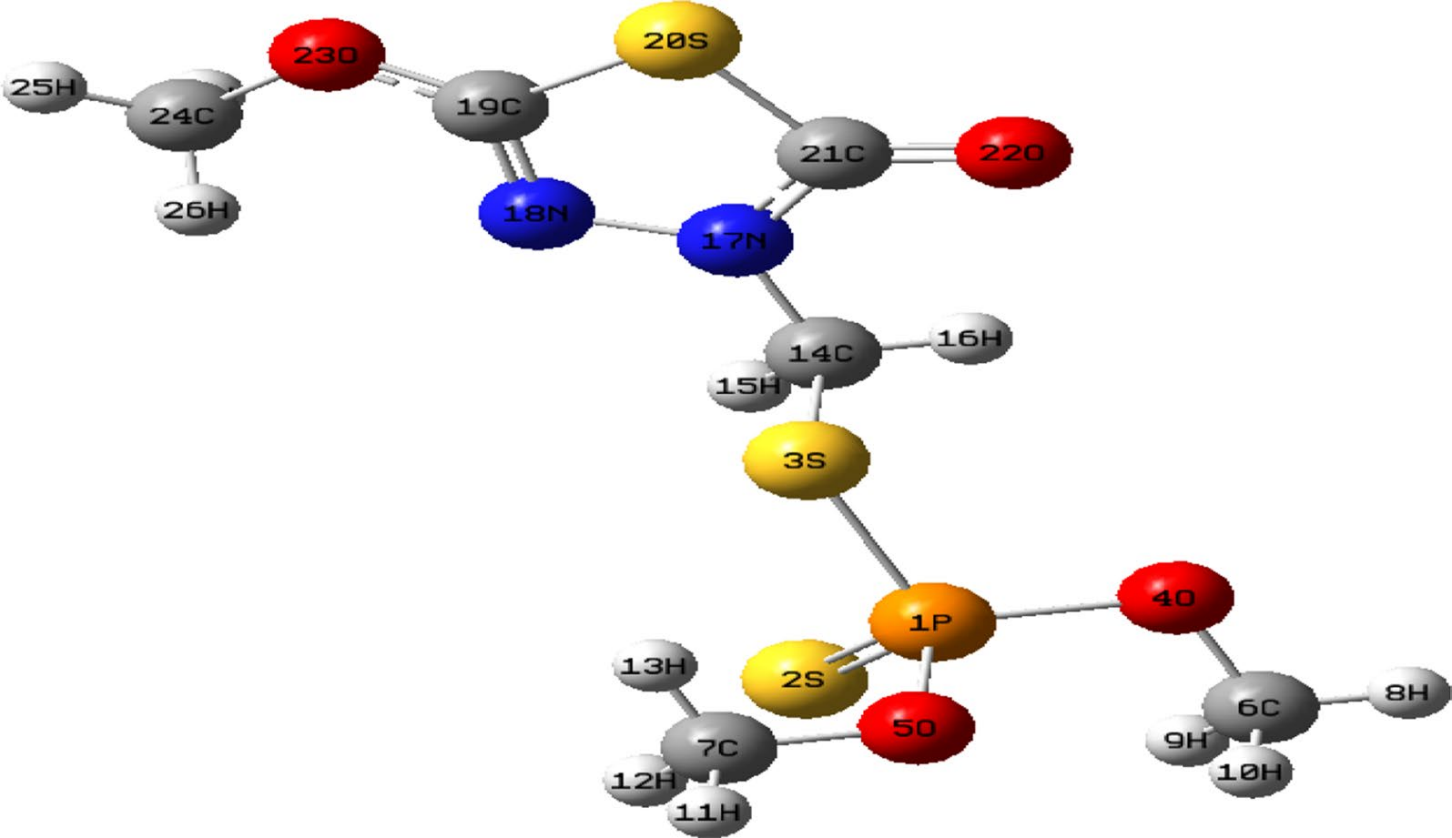
Chemical structure with the atom numbers used in the molecular orbital calculation

According to frontier electron density theory, the calculation of frontier electron density was interesting. The primary position for hydroxyl radical ($\left( {{\text{OH}} \cdot } \right)$ attacked the atoms with the largest electron density, which presented the highest reactivity [16, 17]. In Méthidation, C14, S3 and P1 are the atoms bearing the high electron density, the primary radical attack of $\left( {{\text{OH}} \cdot } \right)$ on C14 should direct with the rupture of the bond C14-S3. The products obtained were not detected under our experimental conditions. Could this absence of detection be due to the high reactivity of radicals $\left( {{\text{OH}} \cdot } \right)$. A new attack was possible in P1 allowing the rupture of the bond P1-S3. Figure 7 shows a chemical structure with the atom numbers used in the molecular orbital calculation.

## Conclusion

Electrolysis of Methidathion was conducted using the two electrodes BDD and SnO_2_, it was performed using same conditions for the two electrodes, namely the same parameters which had been optimum for the preceding experiments. These parameters include the density of current (60 mA/cm^2^), concentration of the electrolyte support (2 %) and the temperature which generates a good effectiveness of the electrodes (T = 25 °C). The comparison of the results relating to the two electrodes indicates that these materials have a power to reduce the quantity of the organic matter in the electrolyzed solution. But the speed of oxidation of these compounds is different according to the materials of the electrodes used. Results showed that, the concentration of the COD decreased exponentially during the time of electrolysis. This could be related to the direct oxidation with the generated hydroxyls radicals. It arises from this comparison, that the electrode BDD is more effective than SnO_2_ for the electrochemical degradation of Methidathion and for the quantity of energy consumed.

Frontier densities were also calculated, results indicated the preferential positions of the attack on Méthidation by the hydroxyls radicals $\left( {{\text{OH}} \cdot } \right)$. And also the calculation of the partial charges indicated that organic molecules produced form oxidation are trapped on the surface of the electrode.

## Abbreviations

BDD: boron doped diamand
SDE: saturated calomel electrode
COD: chemical oxygen demand
CVD: chemical vapor deposition
PGP: potentiostat/galvanostat
